# Children-specific environmental protection strategies are needed in China

**DOI:** 10.1016/j.eehl.2025.01.001

**Published:** 2025-01-09

**Authors:** Jun Zhang, Wen Jiang, Fangbiao Tao, Guodong Ding, Fei Li, Ying Tian, Shu Tao

**Affiliations:** a Ministry of Education-Shanghai Key Laboratory of Children's Environmental Health, Xinhua Hospital, Shanghai Jiao Tong University School of Medicine, Shanghai 200092, China; b School of Public Health, Shanghai Jiao Tong University School of Medicine, Shanghai 200025, China; c School of Public Health, Anhui Medical University, Hefei 230032, China; d College of Urban and Environmental Sciences, Peking University, Beijing 100871, China

**Keywords:** Environmental exposure, Child health, Prevention strategy, China

## Abstract

China, home to over 250 million children, has witnessed remarkable economic development in recent decades, successfully addressing many issues related to basic hygiene and sanitation in children, thereby altering the childhood disease spectrum. However, the emergence of environment-related disorders among children has become a significant concern. Despite the rapid accumulation of scientific knowledge on the adverse effects of environmental pollution on child health, the availability of children-specific protective strategies and actions remains alarmingly low. This commentary synthesizes the information and viewpoints presented and discussed by experts at the International Forum on Children's Environmental Health in China. It summarizes the strategies and actions proposed to reduce adverse environmental exposure and protect children's short- and long-term health and a call for more children-centered evidence-action transformation. The following four specific actions were proposed: (1) strengthen health education in parents, caregivers, and children, and personal protection for children; (2) monitor child exposure and environment-related health status; (3) set up child-specific interventions and regulations; and (4) conduct more research on environment exposures and child health.

## Graphical abstract

**Figure:**
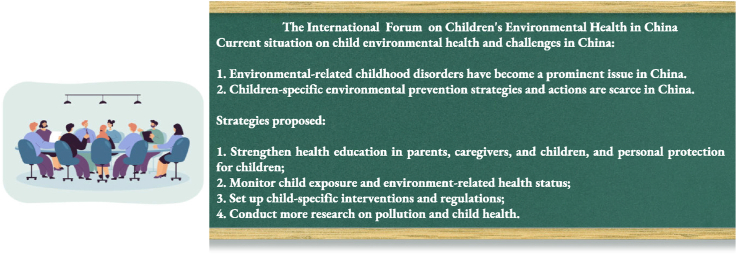

## 1 Introduction

Environmental conditions are essential factors in human health, especially for children. According to the World Health Organization (WHO) estimation, environmental risks (e.g., air pollution, inadequate water, hazardous chemicals, and climate change) account for 25% of disease burdens in children under five years old, and 1 in 4 child deaths could be avoided by reducing these risks [1]. The vulnerability of children to environmental exposures is due to the immaturity in organ development and metabolomic function, consumption of more water and specific food on a per-kilogram body-weight basis, ventilation at a higher rate and lower respiratory zone, and larger surface area-to-volume ratios and higher frequency of hand-to-mouth activity compared to adults, posing them under higher levels of environmental exposure and disease susceptibility [2]. Additionally, early-life environmental exposures could have long-lasting impacts on adolescent and adult health by affecting fetal programming [3]. Thus, implementing child-centered environmental protection measures is vital in protecting long-term population health and achieving sustainable development of society.

China is one of the largest developing countries in the world, with 1.4 billion citizens, of whom over 250 million (18%) are children (0–14 years old) (www.stats.gov.cn). Its remarkable socioeconomic development in the past decades has greatly decreased newborn mortality and solved many problems related to basic hygiene and sanitation in children [4]. However, rapid industrialization and urbanization have also brought new environmental concerns about their contribution to the epidemiological transition towards non-communicable diseases that account for 50.3% of all-cause disability-adjusted life-years among children and adolescents aged 0–19 years in China [5]. Specifically, energy consumption from fossil fuel combustion has exposed 81% of the Chinese population to much higher air pollutant concentrations than the recommended levels set by the WHO, especially for fine particulate matter (PM_2.5_) and ozone in the past decade [[6], [7], [8]]. Besides, as one of the world's largest chemical producers and users, many emerging chemicals with limited safety and toxicity information are already widely used in commerce [9], and some of them (e.g., phenols) are widely detectable in environmental matrices and the general population [10]. Moreover, continuous carbon dioxide and methane emissions from burning fossil fuels and other human activities have increased the number, duration, and intensity of extreme weather conditions and accompanying environmental changes, such as floods and droughts [11,12]. All these environmental risks have contributed to a rising prevalence of child chronic and disabling diseases (e.g., asthma, obesity, neurodevelopmental disorders, cancer, and early puberty) [[13], [14], [15]], possibly by exacerbating inflammation and oxidative stress and disturbing metabolic and hormone homeostasis [13,16].

Despite growing awareness of the threats of environmental risks to child growth and development, child-specific environmental prevention strategies and actions remain scarce. For instance, the Healthy Children Action Enhancement Plan (2021–2025) emphasizes child nutrition, planned immunization, psychological health, and critical illness [17]. Although it has generated many positive impacts, it is short on children's environmental health, which should be integral to child health.

To comprehensively review the current situation on child environmental health and challenges, the Ministry of Education Key Laboratory of Children's Environmental Health at the Shanghai Jiao Tong University School of Medicine, in collaboration with the Chinese Center for Disease Control (CDC) and Prevention and the United Nations Children's Fund (UNICEF) China, hosted the International Forum on Children's Environmental Health in China in Shanghai on October 20, 2023. The objectives of the Forum were to review the current situation of child environmental exposure in China, provide epidemiological evidence on the effects of environmental exposure on child health, and propose child environmental health surveillance and preventive strategies. This commentary synthesizes the information and viewpoints presented and discussed in the Forum. It proposes the following strategies to reduce child environmental exposure and protect their short- and long-term health.

## 2 Proposed strategies

### 2.1 Strengthen health education and personal protection

To protect children from ubiquitous and diverse environmental exposures, government and regulations alone cannot solve the problem. Individual, family, school, hospital, and community efforts based on adequate knowledge of common pollution sources, corresponding health impacts, and prevention methods are also indispensable. Indeed, abundant scientific evidence already exists on the health impacts of some environmental exposures (e.g., heavy metals and air pollution) [18]. Still, effective ways to deliver the messages to the public remain the key obstacle. Thus, educational content targeting common and emerging environmental risk factors should be added to the existing hygiene courses in schools. Schools and communities can also organize regular thematic activities (e.g., knowledge contests) to increase the awareness and interest of children and parents regarding environmental health. Moreover, with increasing informatization, local governments, departments, schools, and communities can take full advantage of social media, such as WeChat official accounts, short videos, advertisements, and network news, to post child environmental health content. All these activities can increase public accessibility to information on child environmental health, help individuals identify potential environmental risks, and lower harmful exposures. For instance, by enforcing bans on tobacco advertising, warning about the dangers of tobacco through media, and advocating tobacco-free families and schools, the prevalence rates of ever-cigarette smokers, current smokers, and frequent smokers among Chinese male secondary school students in 2021 decreased by 10.8%, 26.0%, and 34.4%, respectively, compared to 2019 [19]. For medical training, establishing a new subspecialty of environmental pediatrics and a corresponding fellowship training program may improve pediatricians' awareness of child environmental health. The U.S. Environmental Protection Agency has successfully implemented such a program since 2004, and it can be used as a model [20].

Improving health literacy alone is insufficient. Cultivating good habits and changing unfavorable behavior is the key to implementing effective prevention. Parents and guardians play a critical role in children's environmental health. In early childhood, parents need to recognize potential harmful sources and act to protect children. As children grow up, it becomes important to teach them to protect themselves from environmental exposure and provide timely feedback. All these actions rely on parents acquiring adequate knowledge of children's environmental health through appropriate health education. More importantly, given that many environmental exposures (e.g., PM_2.5_ and chemical contaminants) or triggered endogenous reactive factors can cross the placenta [[21], [22], [23]] and enter breast milk [24] to affect the early-life health of the offspring, the cultivation of maternal healthy behavior during pregnancy and breastfeeding is also beneficial. For instance, pregnant women could decrease the risk of fetal exposure to chemical pollutants, air pollution, and heatwaves by avoiding the usage of cosmetics that contain heavy metals and parabens [25], reducing the intake of certain marine products that have a high level of mercury, perfluoroalkyl substances, and other persistent organic pollutants [[26], [27], [28]], and installing air purifiers and air conditioners.

### 2.2 Monitor child exposure and environment-related health status

Establishing a children-specific surveillance system of significant environmental risks can help estimate the degree of exposure burden in children in time, identify risk sources and main exposure pathways, and perform effective interventions. Schools are an ideal site for monitoring as children spend a large proportion of their time there. China has tried establishing a school-based child health monitoring system and promoting services in the past decades [29]. However, the system is primarily infectious disease prevention-driven and only monitors certain elements of the physical environment (e.g., classroom light, classroom microclimates, drinking water quality, and school canteen hygiene) [30], where many environmental risks that undermine children's health in a more subtle way (e.g., indoor air pollution and chemical contaminants) were not included. Besides, a recent national survey showed that the school's physical environmental monitoring coverage is merely 24.9% and varies greatly between urban and rural areas [31]. Therefore, expanding the coverage and monitoring spectrum to include important environmental factors is needed. A list of major environmental risks for children in schools based on existing research evidence and sampling of food, water, and building materials are first needed, and active surveillance targeting these factors should be then regularly performed to ensure environmental safety. Similar work has been carried out by the European Pediatric Association and collaborating institutions to develop a monitoring system of endocrine-disrupting chemicals in childhood foods and is worthy of reference [32].

Establishing an environmental health index to capture the overall health effects of multiple exposure risks in children can be a helpful step. For instance, based on the nationwide air pollution and mortality monitoring datasets, a research group successfully constructed a national Air Quality Health Index (AQHI) that performs well in predicting daily mortality risk for exposure to air pollutants in China [33]. UNICEF China and the Chinese CDC have been working jointly on a child environmental health index, with more environmental factors (e.g., temperature) involved and targeting specific diseases, which may have significant policy implications.

### 2.3 Set up child-specific environmental interventions and regulations

Regulation and enforcement are two effective ways to prevent environmental exposure. The Chinese government has devoted its efforts to these aspects [34,35]. For instance, the China National Center for Food Safety Risk Assessment has completed a project to characterize the traditional and emerging contaminations of high concern in foods (e.g., bisphenol compounds, ethyl carbamate, and polycyclic aromatic hydrocarbons) and establish specific health-based guide values, which will be used as the direct scientific basis for food safety management policies and regulations [36]. Nonetheless, most exposure limits are not set for children, which may lead to the situation that harmful exposures below the safety limit of adults may still impair children's health. For example, ample evidence has demonstrated that at an environment-relevant exposure dose, many pollutants adversely affect the nervous system in young children but have fewer effects on adults [37]. Thus, for children-sensitive environmental risks, child-specific limits are necessary. Banning Bisphenol A use in infant milk bottles sets an outstanding example [38]. Unfortunately, such an exemplary action is too rare and far from enough to protect child health in the current environment. Furthermore, setting a higher standard for market access to children-related commodities and more severe punishment measures for illegal acts causing children exposure risks could also be an efficient way to reduce harmful exposure.

Building child-friendly cities is another approach to reducing child environmental exposure at the city and community levels. As many Chinese cities are experiencing renovations and new construction, emphasizing child-centered environmental safety from design to construction can significantly benefit child health. In addition to reasonable urban planning to decrease environmental risks (e.g., setting child living, education, and entertainment zones away from the industrial districts, using qualified construction and decoration materials, and providing indoor activity room against extreme weather conditions), expanding urban green and blue spaces coverage and accessibility should also be emphasized, as growing evidence has suggested beneficial effects of these environmental factors on child physical and mental health [[39], [40], [41], [42]].

Lastly, children's environmental health issues involve many aspects that are managed by various government agencies. Key policymakers must coordinate their efforts and take an integrated approach to solving these problems. Detailed responsibility and accountability of individuals and sectors should be clarified to ensure continuous progress.

### 2.4 Conduct more research on environmental exposures and child health

Scientific evidence is the basis of action on potential influential environmental exposures on child health. However, it is well-recognized that our understanding of the field is only the tip of the iceberg. This is because the health effects of many environmental factors, especially ever-growing new chemical contaminants, remain unexplored. Besides, most existing exposure and effect estimations are proxy (vs direct measurements), cross-sectional (vs longitudinal), independent (vs mixture), and incomprehensive (vs exposome), which makes the exposure assessment inadequate [43]. For instance, the normalized difference vegetation index has been widely used in epidemiological studies to represent greenspace coverage. However, the indicator cannot capture the variety and quality of greenness, which has been shown to influence the beneficial effect [44]. As a result, more research with exact and multi-dimension exposure measurements on child environmental health is warranted.

Identifying child health-associated environmental factors alone is only the first step in taking knowledge-action transformation. Evidence on the contribution of the exposure to the disease burden, as well as the accessibility, feasibility, cost-benefit, and cost-effectiveness of the interventions is also needed. Establishing a communication platform that involves research institutions, CDC, and government sectors is encouraged to translate scientific findings into policies more effectively. The platform can take the province as a unit to form a national network. Each county or district CDC can play a pivotal role in connecting schools, researchers, and policymakers to solve outstanding regional child environmental health problems, and provincial CDCs collect local data to form a national database and a network of knowledge translation and sharing.

## 3 Summary and conclusions

China is facing a declining fertility rate and complex environmental challenges. Children carry the nation's future, and their health will be a central concern for families and society and deserve more commitment. It is indeed a daunting task to protect children from environmental risks in their daily lives. However, history has shown that China can do more in this aspect. For example, in 1999, China's State Council issued an act to implement the unleaded gasoline policy nationwide. The action effectively reduced children's geometric mean blood lead level by more than half (from 101.7 μg/L in 1997–2001 to 42.4 μg/L in 2012–2017) [45], one of the triumphs in public health measures in Chinese history.

In the Forum, experts agreed that impactful public health education, comprehensive environment risk monitoring systems, child-specific environmental interventions and regulations, and scientific research evidence are key elements to protect child environmental health in the future. The strategies face the challenges of regional imbalance and continuous human, physical, and financial resource investment, including training scientific and management personnel, maintaining and updating the monitoring system, and stable funding support. Setting up a monitoring and evaluation system is crucial for assessing the progress and effectiveness of the new policies, which can guide further actions and adjustments. As child environmental health is a very complex and constantly changing issue, a long-term comprehensive plan is necessary. The costs will be paid off by decreases in premature deaths and healthcare expenditures on environment-originated diseases and, in the long run, by increases in labor productivity and other social values created by children [46], which is precious fuel for the country's continuous development.

## CRediT authorship contribution statement

**Jun Zhang**: Conceptualization, Writing–Original Draft, Writing– Review & Editing. **Wen Jiang**: Recording, Writing– Original Draft, Writing–Review & Editing. **Fangbiao Tao**: Writing–Review & Editing, Validation. **Guodong Ding**: Writing–Review & Editing, Validation. **Fei Li**: Writing– Review & Editing, Validation. **Ying Tian**: Review and validation. **Shu Tao**: Writing–Review & Editing.

## Declaration of competing interest

All other authors declare no competing interests.
